# Glycidate as a High-Strength Epoxy Adhesive Curable with Amine under Ambient Conditions

**DOI:** 10.3390/polym14050957

**Published:** 2022-02-27

**Authors:** Bungo Ochiai, Katsutaka Soegawa

**Affiliations:** Graduate School of Science and Engineering, Yamagata University, Jonan 4-3-16, Yamagata 992-8510, Japan; soegawaka@gmail.com

**Keywords:** epoxy, amine, glycidate, curing, adhesive, cross-link, DSC

## Abstract

This paper reports that glycidates bearing epoxy moieties with adjacent ester can be cured with diethylenetriamine (DETA) under mild conditions and exhibit high adhesiveness. Curing of bifunctional glycidates with DETA gave cross-linked products. The curing started at a lower temperature (7 °C) than the analogous glycidyl ether (27 °C), while the rate of the curing was slower due to the lower activation energy (*E*_a_ = 57 kJ/g) and exothermicity (Δ*H* = 58 J/g) as confirmed by DSC analysis. The curing system of neopentyl glycol diglycidate and DETA effectively adhered aluminum plates by curing at 25 °C, and the strength was more than five times higher than the curing with analogous glycidyl ether. The higher adhesive strength under curing of ambient conditions and facile preparation of monomers are the significant advantages of this curing.

## 1. Introduction

Epoxy resins are widely applied as high-performance thermosetting resins, and their properties may be tuned by an appropriate choice of curing agents [1,2,3,4,5,6]. The amine curing system is representative and is widely used for adhesives, coating, composites, insulators, etc., due to the robustness of the cured materials and quick curing under mild conditions. Epoxy resins with various structures are commercially available, and most of them are based on glycidyl ether structures. However, glycidyl ethers are of concern because of their toxicity to the environment and living organisms [7,8,9,10], and the development of alternatives is demanded. Other basic skeletons of epoxy resins involve glycidyl esters [11,12,13], glycidyl carbamates [14,15,16], and alicyclic structures [17,18]. Some glycidyl esters commercialized consist of alicyclic structures [6]. The alicyclic epoxy moieties are less reactive than glycidyl ethers, and the ester moieties tend to delay the curing. As a result, these epoxides are advantageous in their longer pot-life, while the curing typically needs higher temperatures and longer times. Glycidyl carbamates are unique epoxides self-cross-linkable under temperature at approximately 140 °C. The cured products are hard, solvent resistant, and flexible [16]. However, these epoxides still cannot solve the problem sufficiently.

We focused on glycidates, epoxides with adjacent alkoxycarbonyl moieties, which are expected to be environmentally degradable. Glycidates can be synthesized by oxidation of acrylates, for which a variety of substrates are commercially available [19,20,21,22,23,24]. For example, we have reported that glycidates can be synthesized from a variety of acrylates using sodium hypochlorite as an oxidant in the presence of quaternary ammonium salts [23]. Although glycidates have been studied for application as intermediates for biologically active compounds [25,26,27,28], little progress has been made in their application to polymers, with the exception of the recent studies on alternating copolymerization with carbon dioxide [29,30]. Therefore, we applied bifunctional glycidates to amine curing, which is the most typical curing system of epoxy resins [3,6]. We report the curing of glycidate with amines that proceeds faster than curing the analogous glycidyl ether under ambient conditions. The higher adhesiveness and lower exothermicity are also the advantages of this system. The curing behavior is discussed in detail based on differential scanning calorimetry (DSC).

## 2. Materials and Methods

### 2.1. Measurements

^1^H and ^13^C nuclear magnetic resonance (NMR) spectra were recorded on a JEOL (Tokyo, Japan) ECX-400 spectrometer (400 MHz for ^1^H and 100 MHz for ^13^C). Fourier transform infrared (IR) spectra were measured by an attenuated total reflection (ATR) mode on a Horiba (Kyoto, Japan) FT-720 spectrometer equipped with a Smiths Detection (London, UK) DuraScope ATR accessory. DSC measurements were conducted on a Seiko Instruments (Tokyo, Japan) DSC6200 instrument at a scanning rate of 10 °C/min under N_2_ flow. Samples were first cooled to −30 °C at 10 °C/min, and measurements were carried out by heating at a scanning rate of 5 °C/min. Tensile tests were carried out on an Imoto Machinery (Kyoto, Japan) IMC-90FE material testing machine equipped with a load cell (20–1000 N).

### 2.2. Materials

Neopentyl glycol diacrylate, 1,9-bisacryloyloxynonane, tetrahexylammonium bromide, and diethylenetriamine (DETA) were purchased from Tokyo Chemical Industry (Tokyo, Japan). Et_2_O, MgSO_4_, hexane, 5% NaOCl aq., and tetrahydrofuran (THF) were purchased from Kanto Chemical (Tokyo, Japan). All the reagents were used as received.

### 2.3. Synthesis of Neopentyl Glycol Diglycidate (NPDG)

Neopentyl glycol diacrylate (2.12 g, 10.0 mmol), tetrahexylammonium bromide (1.4 g, 3.2 mmol), and 5wt% NaOCl aq. (47.6 g) were added in a round-bottomed flask. The mixture was stirred for 1 h in an oil bath maintained at 40 °C. The mixture was transferred to a separation funnel, and Et_2_O (15 mL) and brine (15 mL) were added. The aqueous layer was removed, and the organic layer was dried with MgSO_4_. Then, the mixture was purified by SiO_2_ column chromatography eluted with Et_2_O/hexane v/v (= 2/1). NPDG was obtained as a transparent colorless oil (0.678 g, 2.78 mmol, and 27.8%). ^1^H NMR (400 MHz, CDCl_3_, at rt, *δ* in ppm, *J* in Hz): 1.02 (s, 6H, –C–C*H*_3_), 2.97 (dd, 2H, *J* = 6.4 and 2.8, –O–C*H*_2_–CH–), 2.97 (dd, 2H, *J* = 6.4 and 2.8, –O–C*H*_2_–CH-), 3.46 (dd, 2H, *J* = 6.4 and 6.4, –C*H*–), and 4.02 (s, 2H, –O–C*H*_2_–C(Me)_2_–). ^13^C-NMR (100 MHz, CDCl_3_, at rt, *δ* in ppm): 21.7 (–C–*C*H_3_), 34.9 (–CH_2_–*C*–CH_3_), 46.5 (–O–C*H*_2_–CH–COO–), 47.3 (–O–CH_2_–C*H*–COO–), 69.9 (–O–C*H*_2_–C–), and 169.2 (*C*=O).

### 2.4. Synthesis of 1,9-Nonanediol Diglycidate (NDG)

NDG was prepared in the same manner as that of NPDG using 1,9-bisacryloyloxynonane (2.68 g, 10.0 mmol) in place of neopentyl glycol diacrylate (1.73 g, 5.77 mmol, and 57.7%). ^1^H-NMR (400 MHz, CDCl_3_, at rt, *δ* in ppm, *J* in Hz): 1.26–1.42 (6H, –O–C_2_H_4_–*C*H_2_–*C*H_2_–*C*H_2_–), 1.67 (tt, 4H, *J* = 7.3 and 7.3, –O–CH_2_–*C*H_2_–CH_2_–), 2.97 (dd, 2H, *J* = 6.3 and 2.7, –O–C*H*_2_–CH–), 2.97 (dd, 2H, *J* = 6.3 and 2.7, –O–C*H*_2_–CH–), 3.44 (dd, 2H, *J* = 6.3 and 6.3, –C*H*–), 4.02 (t, 4H, *J* = 7.7, –O–C*H*_2_–C–). ^13^C-NMR (100 MHz, CDCl_3_, at rt, *δ* in ppm): 25.8 (–O–C_4_H_8_–*C*H_2_–), 28.5–29.3 (–O–CH_2_–*C*H_2_–*C*H_2_–*C*H_2_–), 46.3 (–O–*C*H_2_–CH–COO-), 47.4 (–O–CH_2_–*C*H–COO–), 65.8 (–O–*C*H_2_–CH_2_–), and 169.3 (*C*=O).

### 2.5. Curing of Glycidate and DETA (Typical Procedure)

NPDG (244 mg, 1.00 mmol) and DETA (103 mg, 1.00 mmol) were added in a glass vial, and the mixture was stored at 25 °C for 1 h. The gel fraction was calculated by washing the cured product (10 mg) with THF (5 mL) under stirring for 24 h, followed by drying under reduced pressure. 

### 2.6. Tensile Test Procedure

Tensile tests were carried out at a tensile rate of 60 and 5 mm/min for T-peel and tensile shear tests, respectively. The results of three measurements were averaged, and the standard errors are indicated. For T-peel tests, L-shaped aluminum plates (width = 25, thickness = 0.5, length of adhesion part = 50, and length of the part without adhesion = 50 mm) were prepared by bending rectangular plates at a right angle at the center of the long side. For the tensile shear test, rectangular-shaped aluminum plates with a width = 25, thickness = 0.5, and length = 100 mm (adhesion part = 12.5 mm) were used. A mixture of a diepoxide and DETA ([amine]/[epoxy] = 1/1)(approximately 0.1 g) was cast between the adhesion parts using a pipette, and the bonded specimen was cured at 25 °C.

## 3. Results and Discussion

**Scheme 1:**
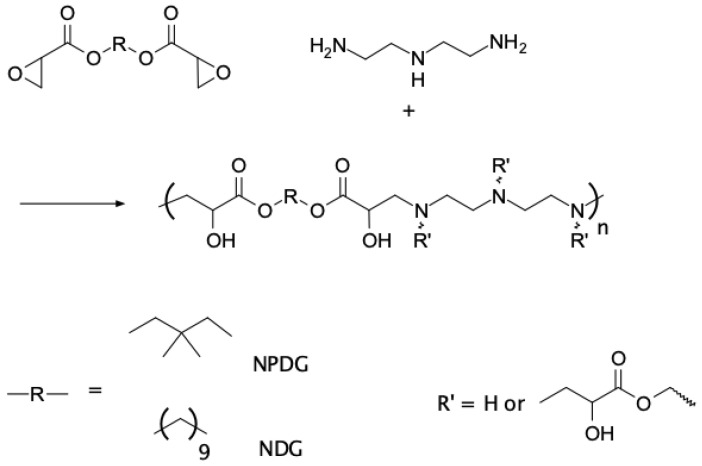
Curing of diglycidate and DETA.

**Figure 1:**
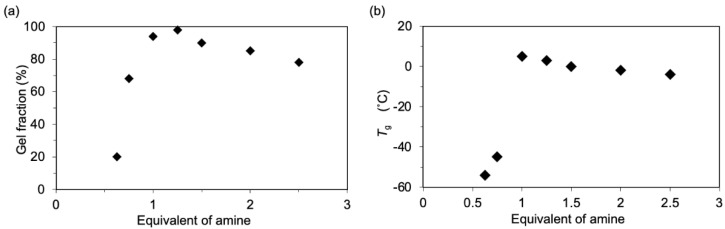
(**a**) Gel fraction and (**b**) *T*_g_ of cured products of NPDG and DETA obtained using different equivalents of amine moieties to epoxy moieties.

The curing reaction was carried out using NPDG and DETA as a curing agent. DETA was added to NPDG, a bifunctional epoxide, under molar feed ratios of the amine-to-epoxy moieties of 0.625, 0.75, 1.0, 1.25, 1.5, 2.0, and 2.5 (Scheme 1, Figure 1). As NPDG was mixed with DETA, a weakly exothermic reaction took place. Then, the mixtures became viscous, and the curing progressed with time. The mixtures were solidified under the conditions of the amine-to-epoxy ratios of 1.0, 1.25, 1.5, 2.0, and 2.5. In contrast, the mixtures remained viscous under the lower amine-to-epoxy ratios of 0.625 and 0.75. The gel fraction became maximum at the [amine]/[epoxy] ratio of 1.25/1.00. The glass transition temperature (*T*_g_) also altered with the [amine]/[epoxy] ratio, and the *T*_g_ became maximum at the feed ratio of 1.0/1.0. The *T*_g_ decreased under the unbalanced feed ratios slightly due to the increase in the number of highly free branched chains. The cross-linking efficiency evaluated from the gel fraction also became higher under the [amine]/[epoxy] ratios close to equimolar. The lower cross-linking efficiency under the lower ratios of amine is ascribable to the reduction in the nucleophilicity of the amine moieties after the addition to the glycidate ring producing electron-withdrawing 2-carboxyalkyl groups. A similar reduction in the reactivity was reported for dual aza-Michael addition of a primary amine group to acrylates in which the reaction rate suddenly dropped at approximately 50% of the conversion [31].

**Figure 2:**
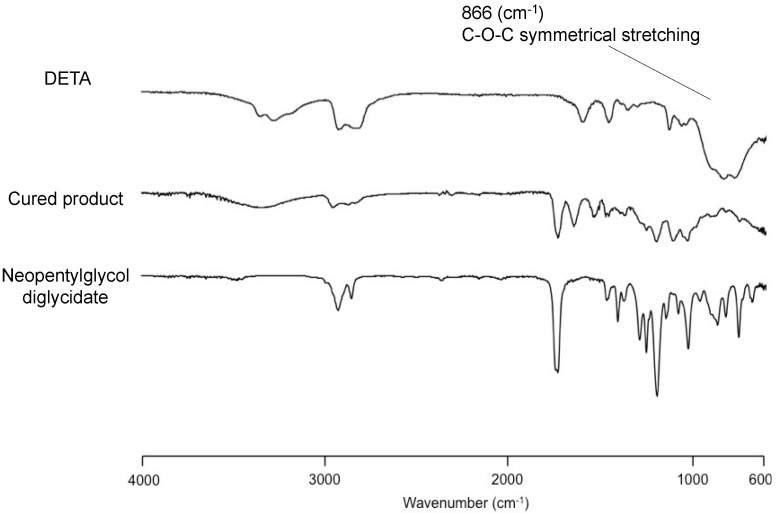
IR spectra of the cured product of NPDG and DETA ([amine]/[epoxy] = 1/1), NPDG, and DETA.

The structure of the product was analyzed by IR spectroscopy (Figure 2). The absorption of the epoxy group at 866 cm^−1^ was not observed in the IR spectrum of the cured product, indicating the complete consumption of the epoxy group by the reaction with DETA. The absorption of the ester carbonyl group was observed at 1732 cm^−1^, and the broad absorption from the O–H and N–H groups produced by the ring-opening was observed in the range of 3000–3300 cm^−1^. These signals agree with the expected structure.

**Figure 3:**
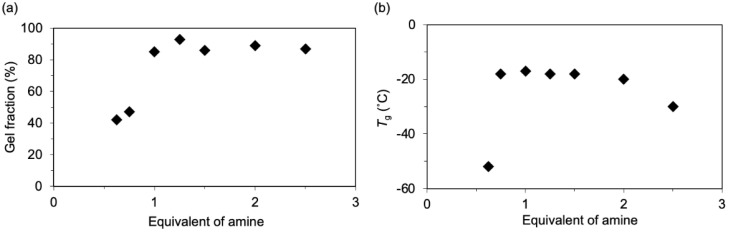
(**a**) Gel fraction and (**b**) *T*_g_ of cured products of NDG and DETA obtained using different equivalents of amine moieties to epoxy moieties.

The curing reaction of 1,9-nonanediol diglycidate (NDG) was also investigated under identical conditions with the amine-to-epoxy molar ratios of 0.625, 0.75, 1.0, 1.25, 1.5, 2.0, and 2.5 (Figure 3). The curing of NDG and DETA was also weakly exothermic, and the mixtures became viscous immediately after mixing. The mixtures were solidified under the conditions of amine-to-epoxy molar ratios of 1.0, 1.25, 1.5, 2.0, and 2.5, while they remained viscous under the amine-to-epoxy ratios of 0.625 and 0.75. The [amine]/[epoxy] ratio for the maximum gel fraction was 1.25/1.00. The gel fractions and *T*_g_ were dependent on the feed ratios as in the case of the reaction between NPDG and DETA. The *T*_g_ of the cured products of NG was lower than those of the cured products of NPDG due to the longer alkyl chain with a higher degree of freedom.

**Figure 4:**
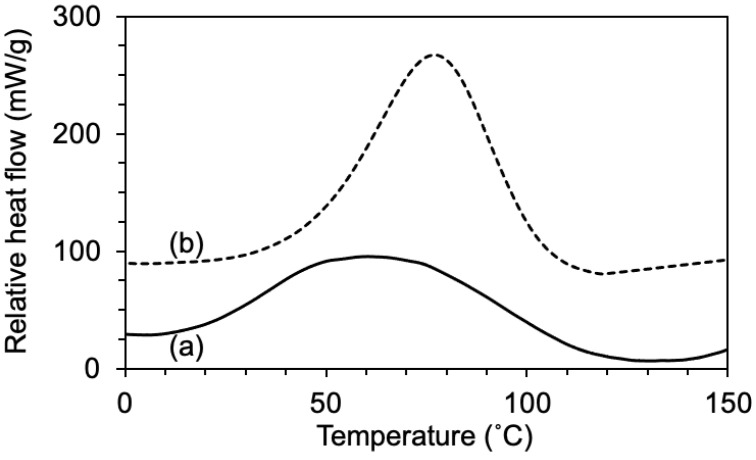
DSC thermograms for curing of (**a**) NPDG and (**b**) NPDGE with DETA (scanning rate = 5 °C/min, N_2_, [amine]/[epoxy] = 1/1).

**Figure 5:**
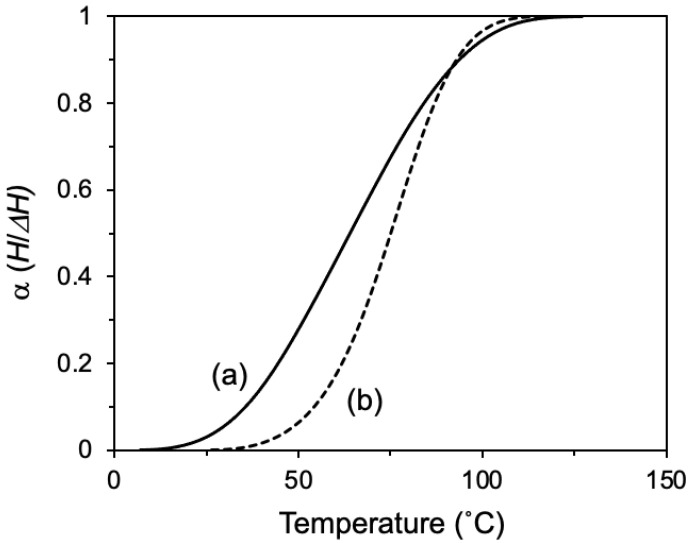
Relationship between integrated exothermic heat α (*H*/Δ*H*) and temperature in the curing of (**a**) NPDG and (**b**) NPDGE with DETA (scanning rate = 5 °C/min, N_2_, [amine]/[epoxy] = 1/1).

The curing behaviors and properties of the cured product of NPDG and DETA were investigated by DSC with the comparison with those of the analogous glycidyl ether, neopentyl glycol diglycidyl ether (NPDGE) [32,33,34]. We employed the products obtained using the equimolar amount of amine and epoxide. Both the curing reactions proceeded efficiently to give highly cross-linked products with high gel fractions. The kinetics of the curing reaction was investigated by DSC (Figure 4 and Table 1). The exothermic peak of the glycidate curing evolved from 7 °C, and its enthalpy was 58 J/g. The curing of glycidyl ether (NPDGE) with a higher enthalpy (82 J/g) started from 27 °C. The lower temperature and lower exotherm of the glycidate system probably originated from the higher reactivity of the epoxy moieties of the glycidate group adjacent to the electron-withdrawing carbonyl group, which increased the susceptibility of the epoxy ring toward the nucleophilic addition of amines. The ^13^C NMR signals of the methylene carbon of the epoxy ring in glycidates appeared at 46 ppm, which is in a lower magnetic field than those in glycidyl ethers that typically appear at 44 ppm, supporting the lower electron density of the epoxy ring in glycidate moieties facilitating the nucleophilic addition of amines kinetically favorable. The progress of the reactions was evaluated by the relationship between the integrated exothermic heat versus temperature (Figure 5). The reaction of NPDG started at a lower temperature, and the reaction rate hardly changed with an increase in temperature. On the contrary, the reaction of NPDGE started at a higher temperature than that of NPDG, but the reaction rate increased with temperature. As a result, the curing of NPDGE ended quickly. A plausible reason is the difference in the spontaneous acceleration, namely, the curing of NPDG with DETA was less exothermic than the curing of NPDGE spontaneously accelerating with larger exothermicity [34]. Typical amine curing of glycidyl ethers does not slow down even at the later stage because the secondary amine structure produced by the first addition is also reactive, and the tertiary amine structure formed by the second addition serves catalytically [6]. On the contrary, the glycidate curing produces amine moieties with lower reactivity resulting in a slower rate at the later stage. The mild progress of the curing of NPDG is advantageous in avoiding the problem of thermal shock led by the high exothermicity. 

**Figure 6:**
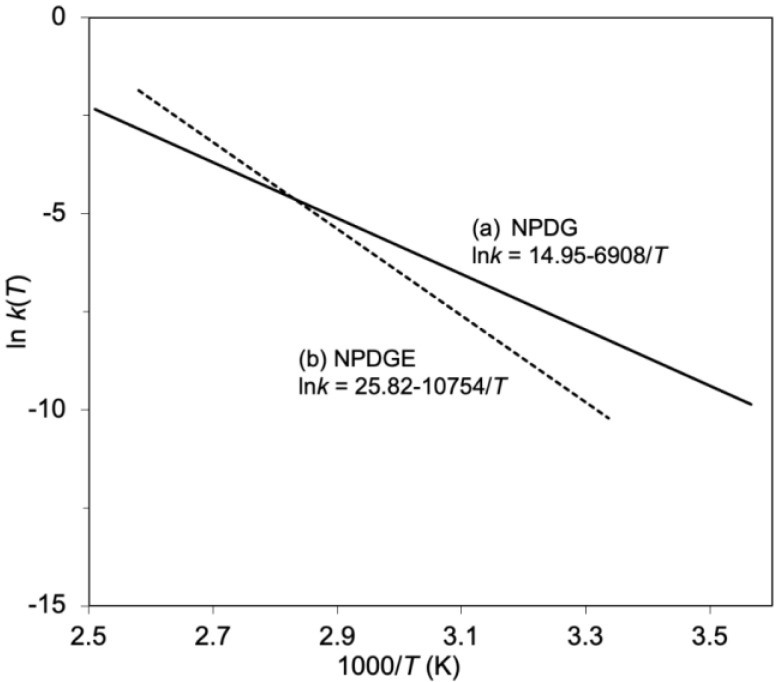
Arrhenius plots of the curing of NPDG and NPDGE with DETA analyzed by DSC (scanning rate = 5 °C/min, N_2_, [amine]/[epoxy] = 1/1).

The activation energies were calculated from the Arrhenius equation (*k*(*T*) = *A*e^−*E*a/*RT*^) based on the method developed by Borchardt and Daniels (Figure 6) [32]. The Arrhenius equations obtained from each plot were: ln*k* = 25.82 − 10754/*T* for NPDGE and ln*k* = 14.95 − 6908/*T* for NPDG.

The Arrhenius parameters were derived from the reaction rate from Equation (1), where d*α*/d*t* is the reaction rate (s^−1^); *α* is the conversion fraction; *k* is the reaction rate constant; *n* is the reaction order (Table 2).

d*α*/d*t* = *kT* (1 − *α*)*^n^* (1)

**Figure 7:**
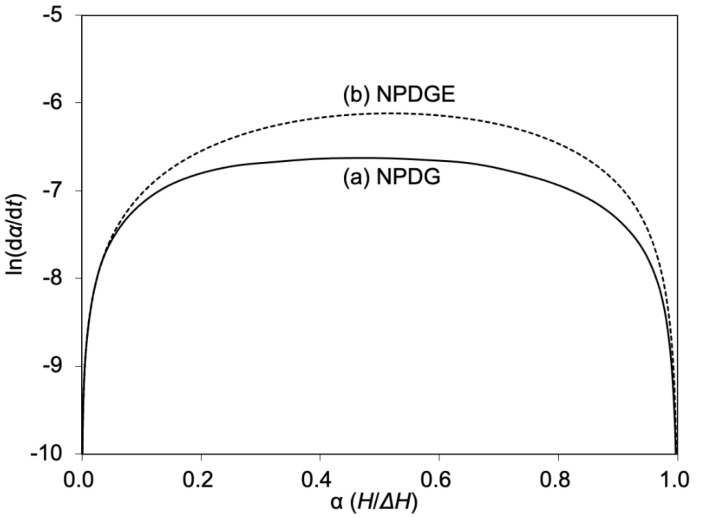
Relationship of ln(d*α*/d*t*) versus *α* (*H*/Δ*H*) for curing of (**a**) NPDG and (**b**) NPDGE with DETA analyzed by DSC (scanning rate = 5 °C/min, N_2_, [amine]/[epoxy] = 1/1).

The relationship between the reaction rate (ln(d*α*/d*t*)) and the conversion calculated from the heat of polymerization (*H*/Δ*H*) is shown in Figure 7. The curve for NPDGE was above that of NPDG due to the spontaneous acceleration during the curing. While the activation energy of the curing of NPDG was smaller, the reaction proceeded slowly throughout the curing due to the lower enthalpic gain. On the contrary, the curing of NPDGE proceeded faster due to the acceleration at the later stage originating from the higher enthalpic gain overcoming the higher activation barrier. These activation energies and heats of curing explain the reaction behaviors well.

**Figure 8:**
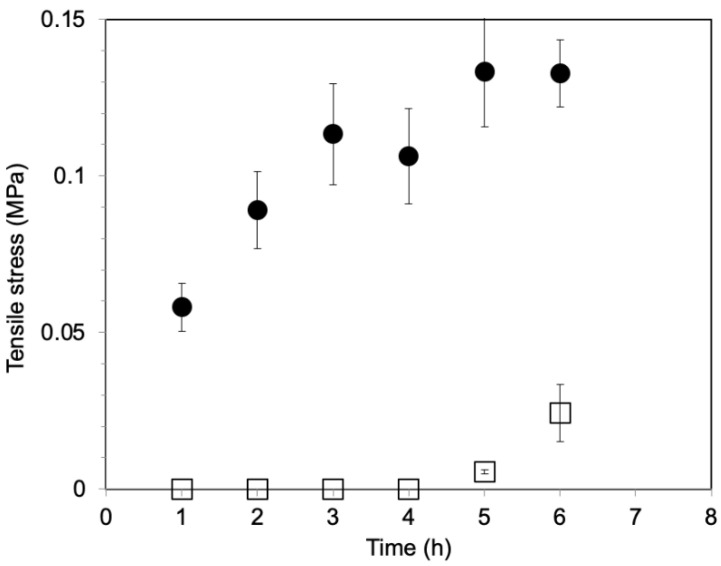
Time course of T-peel tensile stress during adhesion of two L-shaped Al plates at 25 °C with mixtures of NPDG (circle) and NPDGE (square) with DETA (tensile rate = 60 mm/min, adhered part = 25 × 50 mm, [amine]/[epoxy] = 1/1).

**Figure 9:**
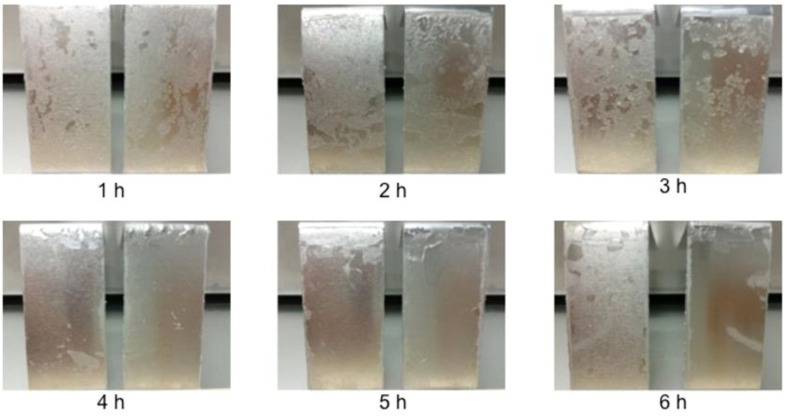
Photo images of surface of two L-shaped Al plates cured with mixtures of NPDG with DETA at 25 °C for 1–6 h peeled by T-peel tensile stress test (tensile rate = 60 mm/min, adhered part = 25 × 50 mm, [amine]/[epoxy] = 1/1).

This epoxy–amine curing system was applied for adhesion. A mixture of a diepoxide and DETA with the equimolar ratio of the epoxy and amine groups was applied on an L-shaped aluminum plate, and another L-shaped plate was bonded at 25 °C. The adhesive strength was measured after the different curing times (1, 2, 3, 4, 5, and 6 h) by a T-peel tensile test (tensile speed = 60 mm/min). The curing with NPDG with DETA showed significant adhesive strength after 1 h, while the curing of NPDGE showed significant adhesion after 5 h (Figure 8). The faster increase in the adhesion of NPDG under the ambient conditions agreed with the faster curing under ambient conditions due to the lower activation energy. Other possible factors of the high adhesiveness were the aforementioned lower exothermicity and the higher polarity originating from the ester group in glycidate than the ether group in glycidyl ether. The strengths increased with time in both curing, while the standard error of the curing of NPDG became larger after 3 h. The peeled surface of the plate adhered with NPDG was observed (Figure 9). In the initial stage, cohesive failure predominated over adhesive failure, indicating the high affinity of the cured product for aluminum. The failure mode shifted to adhesive failure as the progress of the curing. The concomitant occurrence of both adhesive and cohesive failures was a probable factor for the higher standard errors between 3 and 5 h of curing. The NPDG–DETA curing exhibited higher adhesiveness than the conventional NPDGE–DETA curing, implying its potential for a wide range of applications for adhesives, coating, and electric materials.

**Figure 10:**
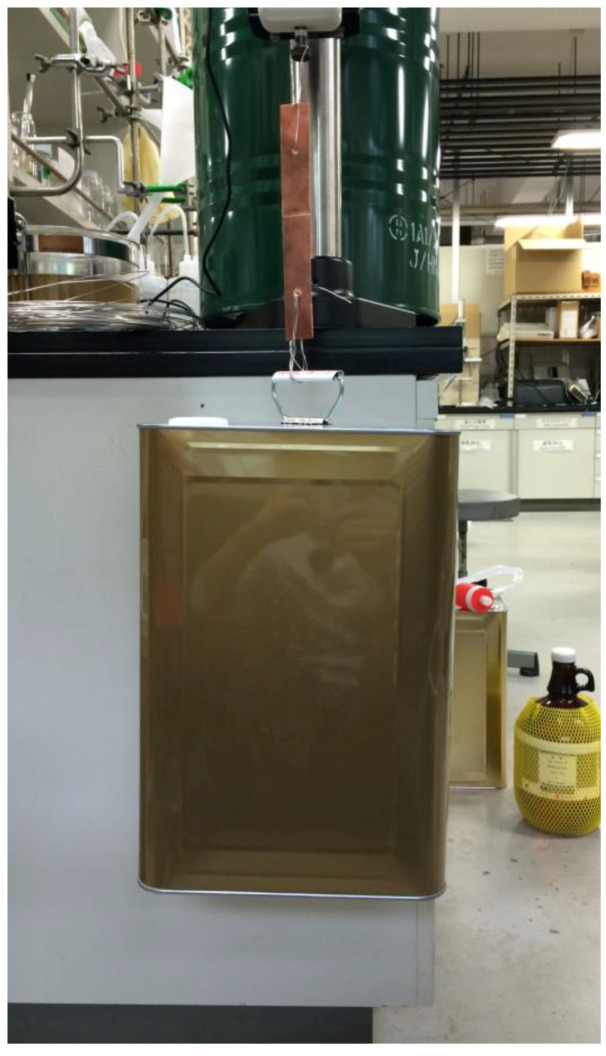
Photo image of aluminum plates adhered with NPDG and DETA (curing time = 6 h, 25 °C, [amine]/[epoxy] = 1/1) loading a can containing 11 kg of hexane.

Tensile shear tests were also carried out. A mixture of a diepoxide and DETA with the equimolar amount of the epoxy and amine groups was applied between rectangular aluminum plates and was cured for 6 h at 25 °C. The adhesive strength was measured using a tensile testing machine (tensile speed: 5 mm/min). The shear stress of the cured product of NPDG (1.14 ± 0.08 N/mm^2^) was much higher than that of the cured product of NPGE (0.03 ± 0.01 N/mm^2^). This result also indicates the excellent adhesiveness with faster curing of the glycidate-based epoxy resin at ambient temperature. In addition, the cured product was so strong that it was not broken even by loading an 11 kg can of hexane for at least 30 min (Figure 10).

## 4. Conclusions

Bifunctional glycidates, easily prepared by aqueous oxidation of diacrylates, can be cured with DETA under mild conditions, and the cured product exhibited high adhesiveness in the adhesion of aluminum plates. Essential features of the glycidate–amine curing are lower temperature for curing, lower exothermicity, and higher adhesive strength than the curing employing analogous glycidyl ether. The lower initiation temperature is advantageous in curing outside in cold places, for example, a connection of water pipes, architectural adhesion, and painting wall in winter. The lower exothermicity is advantageous in smaller dimensional changes originating from changes in temperature. These unique features of glycidates are motivating us for applications of glycidates to other curing systems and investigation on the toxicity and environmental impact of glycidates and their cured products.

## Figures and Tables

**Table 1:** DSC result of curing of NPDG and NPDGE with DETA ([amine]/[epoxy] = 1/1).

Epoxide	Δ*H* (J/g)	*T*_i_ ^1^ (°C)	*T*_p_ ^2^ (°C)	*T*_f_ ^3^ (°C)
NPDG	−58	7	62	127
NPDGE	−82	27	77	116

^1^ Initial temperature of curing; ^2^ temperature of peak top of exothermic peaks; ^3^ final temperature of curing.

**Table 2:** Arrhenius parameters and reaction orders of curing of NPDG and NPDGE with DETA analyzed by DSC (scanning rate = 5 °C/min, N_2_, [amine]/[epoxy] = 1/1).

Epoxide	*E*_a_ ^1^ (kJ/mol)	*A*^2^ (s^−1^)	*n* ^3^
NPDG	57	3.1 × 10^6^	1.3
NPDGE	89	1.6 × 10^11^	1.3

^1^ Activation energy; ^2^ preexponential factor; ^3^ reaction order derived by the least squares from the relations between time and enthalpy.

## Data Availability

Not applicable.
